# Ecological niche and potential distribution of *Anopheles arabiensis* in Africa in 2050

**DOI:** 10.1186/1475-2875-13-213

**Published:** 2014-06-03

**Authors:** John M Drake, John C Beier

**Affiliations:** 1 Odum School of Ecology, University of Georgia, 140 E Green Street, 30602-2202 Athens, GA, USA; 2 Department of Public Health Sciences, University of Miami Miller School of Medicine, 1120 NW 14th Street, 33136 Miami, FL, USA

**Keywords:** *Anopheles arabiensis*, Ecological niche model, Climate change

## Abstract

**Background:**

The future distribution of malaria in Africa is likely to be much more dependent on environmental conditions than the current distribution due to the effectiveness of indoor and therapeutic anti-malarial interventions, such as insecticide-treated nets (ITNs), indoor residual spraying for mosquitoes (IRS), artemisinin-combination therapy (ACT), and intermittent presumptive treatment (IPT). Future malaria epidemiology is therefore expected to be increasingly dominated by *Anopheles arabiensis*, which is the most abundant exophagic mosquito competent to transmit *Plasmodium falciparum* and exhibits a wide geographic range.

**Methods:**

To map the potential distribution of *An. arabiensis* in Africa, ecological niche models were fit to 20th century collection records. Many common species distribution modelling techniques aim to discriminate species habitat from the background distribution of environments. Since these methods arguably result in unnecessarily large Type I and Type II errors, LOBAG-OC was used to identify the niche boundary using only data on *An. arabiensis* occurrences. The future distribution of *An. arabiensis* in Africa was forecasted by projecting the fit model onto maps of simulated climate change following three climate change scenarios.

**Results:**

Ecological niche modelling revealed *An. arabiensis* to be a climate generalist in the sense that it can occur in most of Africa’s contemporary environmental range. Under three climate change scenarios, the future distribution of *An. arabiensis* is expected to be reduced by 48%-61%. Map differences between baseline and projected climate suggest that habitat reductions will be especially extensive in Western and Central Africa; portions of Botswana, Namibia, and Angola in Southern Africa; and portions of Sudan, South Sudan, Somalia, and Kenya in East Africa. The East African Rift Valley and Eastern Coast of Africa are expected to remain habitable. Some modest gains in habitat are predicted at the margins of the current range in South Sudan, South Africa, and Angola.

**Conclusion:**

In summary, these results suggest that the future potential distribution of *An. arabiensis* in Africa is likely to be smaller than the contemporary distribution by approximately half as a result of climate change. Agreement among the three modelling scenarios suggests that this outcome is robust to a wide range of potential climate futures.

## Background

Despite substantial reductions in malaria incidence, particularly in sub-Saharan Africa [1,2], the global burden of malaria remains in the hundreds of millions of cases annually. Recent estimates range from 225 to 515 million cases per year [3-5], resulting in more than a half million deaths per year [4,6]. Although the decline of malaria in sub-Saharan Africa is generally attributed to anti-malarial interventions including the distribution of insecticide-treated nets (ITNs), artemisinin-combination therapy (ACT), intermittent presumptive treatment (IPT, [7]), and indoor residual spraying for mosquitoes (IRS), these cannot account for all observed reductions in malaria incidence [2]. For instance, malaria was observed to decline from holoendemic levels to prevalence of 30% to 50% on the island of Pemba, Tanzania prior to the onset of vector control activities [8]. An entomologic study by Meyrowitsch *et al.*[9] of two rural communities in the nearby Tanga region of Tanzania indicated that these declines are most likely due to declines in the abundance of *Anopheles gambiae* and *Anopheles funestus* mosquitoes, which they in turn attributed to declining precipitation and interruptions in annual (periodic) rainfall patterns [9]. These studies indicate that changes in climate and consequent weather patterns may be equally responsible for declines in African malaria, at least in some regions [9].

Despite decelerations in the emission of greenhouse gases, changes in the global climate system are now widely expected to continue to 2100 and beyond [10]. The potential consequences of these changes for human health, and the distribution of vector-borne diseases in particular, has been especially controversial [1,11-20]. The recent surge in anti-malarial activity has given rise to a new question: What will be the future distribution of malaria, given that both entomologic and epidemiologic circumstances are changing? Among other anticipated effects of these changes, it seems probable that future human cases of malaria in Africa will be disproportionately due to the vector *Anopheles arabiensis* compared with historical patterns because the historically dominant vectors, *An. gambiae* and *An. funestus*, are selectively targeted by indoor interventions [21] and in many cases are declining in relative abundance [9,22,23]. *Anopheles arabiensis*, by contrast, exhibits greater behavioral plasticity, is more associated with outdoor habitats, and is more likely to bite susceptible persons out of doors (exophagy) where protections are less likely to be in place [24]. Particularly, *An. arabiensis* is well known to favour dry (savannah) disturbed habitats [24] while larval habitats are primarily small, temporary, freshwater pools and other built features of the landscape, such as rice fields and fish ponds [24]. Additionally, *An. arabiensis* is more commonly found in urban environments, where an increasing proportion of sub-Saharan Africans reside, than *An. gambiae*[25]. Thus, interventions with ITNs are less effective against *An. arabiensis* than *An. gambiae* and *An. funestus*[26]. Further, because *An. arabiensis* executes its life cycle outside the built environment, it serves as a transmission route more likely to be subject to climate fluctuations. Taken together, these observations suggest that even as the transmission of malaria may be expected to continue to decline (because of aggressive interventions and continuing urbanization of the human population) that portion of transmission that remains will be disproportionately due to *An. arabiensis* and disproportionately subject to environmental conditions, a pattern that has been reported in lowland areas of Nyanza Province, Kenya [27].

Mapping the future potential distribution of *An. arabiensis* is therefore an important step to determining the future geography of malaria. Prior work has focused on modelling the microhabitat conditions conducive to mosquitoes, including land cover and human population and development over smaller areas [28]. In contrast, the expression *potential distribution* is used to refer to the large-scale geographic regions permissive to the persistence of a vector species in the absence of vector control, as set by conditions of the regional climate. That is, the potential distribution concerns not only the most conducive environmental conditions, but also the conditions at its environmental margins. Mapping the probable effects of climate change on the distribution of *An. arabiensis* therefore requires first estimating its *ecological niche*, the range of environmental conditions in which *An. arabiensis* is found. Because of its ecology, the space of these conditions is determined primarily by temperature and precipitation [29].

Many studies of species distributions seek to fit a model that discriminates the environments in which a species is found from the distribution of environments in a representative geographic region (the “background”) [30-32]. Such models, commonly called “presence-background models” may be proportional to the probability of species presence [32-34], but should not be considered models of the ecological niche because niche environments are a subset of the environmental background, not a complementary set, and because not all niche environments are in fact occupied.

**Figure 1:**
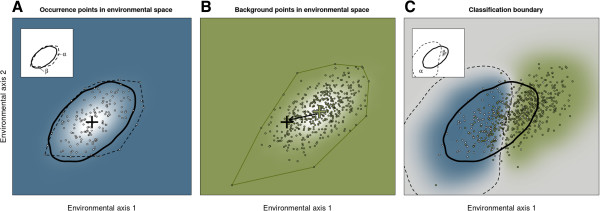
**Boundary estimation versus discrimative methods for ecological niche modelling.** Simulated data illustrates why modelling a species’potential distribution is a problem for boundary estimation not classification. **A**. The left most panel represents the habitat in two environmental dimensions (*e.g.*, precipitation and temperature) in locations at which a species is known to occur. The heavy curve depicts the true niche of the species. The dashed line is the convex hull of the sample, a naive estimate of the species niche. The black cross represents the center of the species niche, which is the most probable set of environmental conditions at which the species occurs. **B**. The center panel represents samples of environmental conditions at locations taken at random from the background distribution of environments. The green cross indicates the mean environment. The arrow is a vector of “niche displacement”. **C**. The right most panel depicts both occurrence and background data. The dashed line is the estimated optimal classification boundary between occurrence and background points. The blue-green color gradient depicts the conditional probability that a given instance is an occurrence points (blue: *P* (*occurrence*) = 1; gray: *P* (*occurrence*) = 0*.*5; green: *P* (*occurrence*) = 0). Inset plots illustrate the region of environmental space in which each fit model makes Type I (*α*) or Type II (*β*) errors.

A hypothetical scenario illustrates the problem (Figure 1). In each of three panels are two hypothetical environmental variables. In the left most panel are points which represent sampled environmental conditions at which the species is found. These points are sampled from a bivariate Gaussian density. The bold line corresponds to probability density *p =* 0*.*002, and represents the “true” niche boundary. The true population mean of this density is indicated by the black cross. The convex hull of these points is also drawn as a “naive” model of the niche. Insofar as this naive model does not coincide with the true niche boundary it makes Type I (*α*) and Type II (*β*) errors (inset plot). In the centre panel, plotted on the same axes, are samples of the bivariate Gaussian distribution of environmental conditions from the “background” distribution. For comparison with the first panel, a convex hull is again plotted around the set of observed background points. The true mean of this density is indicated by a green cross. The displacement between the black cross and the green cross shows that this hypothetical species is in fact selective for particular environments – it is not simply found in environments in proportion to their realization in nature. Finally, both sets of points are plotted together in the third panel. Additionally, the conditional probability that an observation sampled from any point in the space would be an occurrence or background instance is plotted with a blue-green gradient. The classification boundary optimal for separating occurrence and background points (*i.e.*, *P* (*occurrence|x*_1_*, x*_2_) = 0*.*5)) is shown with a dashed line. This classification boundary is representative of the kind of model fit by presence-background estimators. The inset plot depicts the Type I and Type II errors associated with this model, clearly much larger than for the naive convex hull model.

Inspection of the figure shows why this is so: since the occurrence data are a subset of the background, the classification boundary is biased in the direction of the displacement between the niche and the background. Intuitively, one can see there are two reasons why this boundary must be biased and why the errors must be distributed in the (*x*_1_*, x*_2_) coordinate space in the way that they are: (1) Extreme environments in the direction of niche displacement will be assigned to the niche even when there is no evidence that these environments belong to the niche; (2) Intermediate environments in the opposite direction will be incorrectly classified as unsuitable, despite being nearly central within the niche, because the relatively frequency of these points is small compared with the vast number of background points presented to the model. What this illustration shows is that to get a good model (dashed lines) of the true niche (heavy curve) requires drawing a boundary that is, in some sense, “around” the observed occurrence points, not one that discriminates occurrence points from the background.

In conclusion, this example illustrates why ecological niche modelling should be construed as a problem for *boundary identification*, not *classification*. Indeed, regardless of how effective discriminative methods are at predicting contemporary species collections (*i.e.*, the conditional probability of occurrence), such results should be viewed with caution when it comes to estimating potential distributions and for extrapolating to future climate scenarios, which may contain so-called *no analog environments*, combinations of environmental conditions not presently in existence on earth and therefore not available to learn from.

*Low bias bootstrap aggregation for one class data* (LOBAG-OC) is a recently developed boundary identification method for ecological niche modelling [35]. LOBAG-OC is conceptually superior for this task, compared with habitat suitability models that return a continuous measure such as *Ecological Niche Factor Analysis* (ENFA) [36] or MAXENT [33,37], because LOBAG-OC estimates the niche boundary directly. This is important for two reasons. First, because ENFA, MAXENT, and related methods draw on the higher moments (mean and variance) of the observed distribution of occurrence points, they are least accurate at the range boundaries and vulnerable to biases in sampling. Second, the translation of a measure of habitat suitability into a potential range requires the determination of a “cut-off” that is rarely amenable to empirical analysis and so therefore must be determined more or less arbitrarily. Additionally, in comparative tests, LOBAG-OC has been shown to outperform other popular boundary identification methods [35] such as BIOCLIM [38] and DOMAIN [39].

LOBAG-OC was used to fit an ecological niche model to 20th century point occurrence data on the distribution of *An. arabiensis* in Africa. This analysis showed that, despite its lower prevalence compared with *An. gambiae*, *An. arabiensis* is nevertheless a climate generalist in the sense that it tolerates a wide range of climate conditions. The fit model was then applied to simulated data for three climate change scenarios to forecast the future potential distribution of *An. arabiensis*. Specifically, the fit niche model was evaluated on maps of climate projections for the year 2050 generated by the Hadley CM 3 model for scenarios A1B, A2A, and B2A. These scenarios derive from the Intergovernmental Panel on Climate Change (IPCC) Special Report on Emissions Scenarios [40]. Scenarios reflect political story lines that emphasize policies aimed at economic considerations (Scenarios A1B and A2A) or environmental protection and social equity (Scenario B2A). All scenarios produce ranges for projected global surface warming by 2100 that are intermediate among the scenarios considered by the IPCC [10]. Scenario A1B describes a future world of rapid economic growth, a global population that peaks in mid-century, and rapid introduction of more efficient energy technologies. Scenario A2A envisions a more heterogeneous future world where economic growth and technological change are slower and unevenly distributed. Scenario B2A is a world with more local approaches to environmental sustainability, yielding a slowly increasing global population, intermediate levels of economic development, and less rapid and more diverse technological change than in the A1 scenario [40].

Our model predicts that if any of these three climate change scenarios is realized, the result will be significant reductions in the total land area hospitable to *An. arabiensis*. A comparison of modeled current distribution and forecasted potential distribution identified regions where *An. arabiensis* habitat is lost and regions where it is gained. Map intersections for the three climate change scenarios show that these results are robust to a wide range of assumptions about the future climate system.

## Methods

### Data

**Figure 2:**
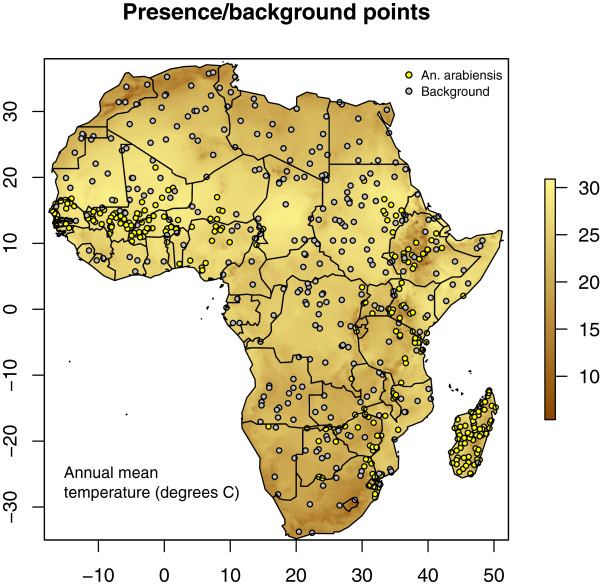
**Spatial distribution of *Anopheles arabiensis*.** Distribution of sampling points and a balanced random sample of background points.

Occurrence records were obtained by downloading the coordinates of collection sites for *An. arabiensis* from the Mapping Malaria Risk in Africa data clearinghouse [41-43]. These records reflect collections between 1956 and 1996. Duplicate points were removed and the data thinned so that no more than one record was retained within a distance of 0.5 degrees of another point. The length scale of this thinning depends on latitude, but is around 50 km, which is five to ten times the typical flight distance for a mosquito and a greater distance than is likely to be traveled by an investigator to obtain a convenience sample. Probably, the thinned data are not completely spatially independent, but this thinning should have removed the most egregious multiply sampled populations. This procedure yielded *n*_*p*_ *=* 307 presence records. Prior to model fitting, the occurrence points were randomly split into training (80%) and testing (20%) sets, yielding a total of *ñ*_*p*_ *=* 246 observations in the training set. For testing, an additional *ñ*_*p*_ points were randomly selected from the background distribution of environments (Figure 2).

Models were fit to 86 “baseline” environmental features reflecting average conditions for years 1950-2000 (Table 1). Data included 36 interpolated measured variables (average monthly minimum and maximum temperature and precipitation) and the 19 constructed BIOCLIM variables from the WorldClim data set [44]. Raw data for both baseline and forecasted climate were obtained from [45] where they are reported in a 10 minute resolution after statistical downscaling using the delta method [44]. An additional 31 features were constructed, including monthly temperature range, logarithmic transforms of highly skewed variables (monthly precipitation, precipitation in the warmest quarter, and precipitation in the coldest quarter), and empirical cumulative distribution function (ecdf) transforms of some skewed variables (annual precipitation, precipitation in the wettest month, precipitation in the driest month, precipitation in the wettest quarter, and precipitation in the driest quarter). Prior to feature construction, data were clipped to exclude observations not coinciding with continental land regions of Africa or Madagascar. After feature construction, all layers were rescaled by subtracting the mean and dividing by the standard deviation of the baseline data to ensure that both baseline and forecasted data were rescaled to a common range. Features which are ecdf transforms were not rescaled. A principal components analysis was performed to investigate the gross structure of the baseline environmental data.

**Table 1:** **Environmental features used to model the potential distribution of *An. arabiensis *** **in Africa**

**Description**	**Units**	**Number of variables**
*Measured variables*		
Average monthly minimum temperature	°C	*n =* 12
Average monthly maximum temperature	°C	*n =* 12
Average monthly precipitation	°C	*n =* 12
*BIOCLIM variables*		
(BIO1) Annual mean temperature	°C	*n =* 1
(BIO2) Mean diurnal temperature range	°C	*n =* 1
(BIO3) Isothermality	no units	*n =* 1
(BIO4) Temperature seasonality	°C	*n =* 1
(BIO5) Maximum temperature of warmest month	°C	*n =* 1
(BIO6) Minimum temperature of coldest month	°C	*n =* 1
(BIO7) Temperature annual range	°C	*n =* 1
(BIO8) Mean temperature of wettest quarter	°C	*n =* 1
(BIO9) Mean temperature of driest quarter	°C	*n =* 1
(BIO10) Mean temperature of warmest quarter	°C	*n =* 1
(BIO11) Mean temperature of coldest quarter	°C	*n =* 1
(BIO12) Annual precipitation	mm	*n =* 1
(BIO13) Precipitation of wettest month	mm	*n =* 1
(BIO14) Precipitation of driest month	mm	*n =* 1
(BIO15) Precipitation seasonality	mm	*n =* 1
(BIO16) Precipitation of wettest quarter	mm	*n =* 1
(BIO17) Precipitation of driest quarter	mm	*n =* 1
(BIO18) Precipitation of warmest quarter	mm	*n =* 1
(BIO19) Precipitation of coldest quarter	mm	*n =* 1
*Constructed features*		
Monthly temperature range	°C	*n =* 12
log-transforms	log mm	*n =* 14
ecdf-transforms	no units	*n =* 5

### Model fitting

Niche modelling was performed using the LOBAG-OC algorithm, a computational approach for modelling ecological niches from presence-only data [35]. LOBAG-OC is an ensemble learning approach for one-class-classification that averages the outcomes of a large number of weakly regularized one-class support vector machines to obtain a numerical value for any given combination of environmental inputs [35]. Briefly, the model fitting algorithm iteratively resamples the original data, fits a one-class support vector machine to this sample (a machine learning approach to estimating the support of a statistical distribution, referred to as a *base model*), and stores the result. A prediction is made by averaging the predictions of the stored base models. By construction, the method is relatively insensitive to irrelevant data or biased sampling, two features that are important for ecological niche modelling. This model has two tuning parameters, *ν*, which governs the degree of regularization of the base learners, and the number of votes. As a rule of thumb, LOBAG-OC was shown to perform near optimally with only 2^6^ = 64 votes (but has not been found to diminish in performance as the number of votes increases) and in a large neighborhood of *ν* around *ν =* 2^*−*4^[35]. The model reported here was fit using 256 votes and *ν =* 2^*−*4^.

LOBAG-OC modelling provides a nonparametric mappable summary of the ecological niche. Generalized boosted regression models [46,47] were applied to a randomly selected subset of 10,000 locations to investigate the relative importance of constituent environmental variables to determining the boundaries of the *An. arabiensis* geographic range. First, model output was binarized (niche/non-niche) and associations between these labels and 86 environmental covariates were learned using boosted regression trees. The optimal number of trees was selected using fourfold cross-validation. Relative influence was quantified using the method of Breiman [48]. Variables with > 5% relative influence are reported. An additional 10,000 random locations were inspected to identify differences between baseline and projected conditions (*i.e.*, change from niche to non-niche or change from non-niche to niche) for each climate scenario. Associations between these changes and environmental covariates were identified in the same way.

## Results

**Figure 3:**
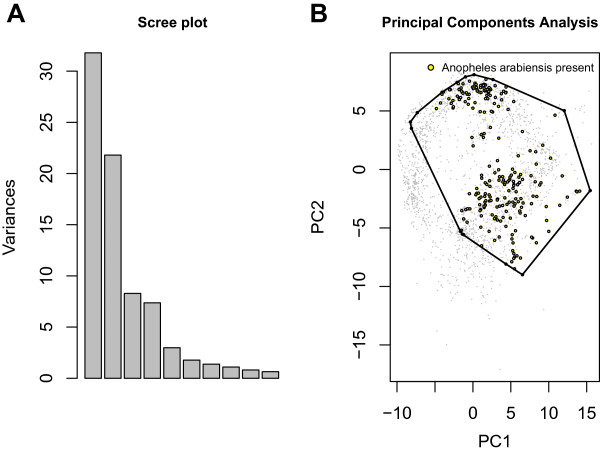
***Anopheles arabiensis *is found across a broad range of environments. A**. Scree plot of the first ten principal components shows that a majority of the environmental variation (≈55%) may be summarized by the first two principal components. **B**. Points where *Anopheles arabiensis* has been collected represented in the space of the first two principal components of the environmental features shows that this species occupies a very large environmental range.

**Figure 4:**
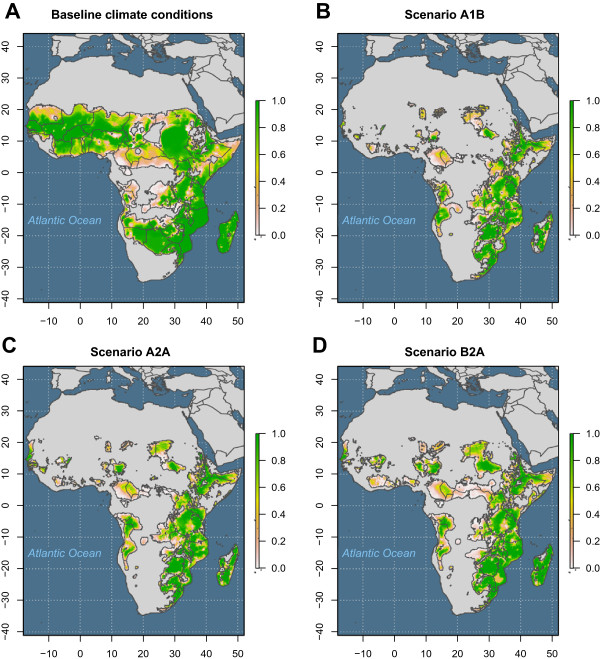
**Potential distribution of *Anopheles arabiensis *under contemporary conditions and three global climate change scenarios. A**. Modelled potential distribution of *Anopheles arabiensis* habitat in Africa given the current global climate. **B**. Future potential distribution of *Anopheles arabiensis* in Africa under IPCC Scenario A1B. **C**. Future potential distribution of *Anopheles arabiensis* in Africa under IPCC Scenario A2A. **D**. Future potential distribution of *Anopheles arabiensis* in Africa under IPCC Scenario B2A.

Principal components analysis showed the environmental space represented by WorldClim to be relatively low dimensional. Particularly, approximately 55% of the variation in environmental covariates is contained in just the first two principal components (Figure 3). Plotting of *An. arabiensis* occurrence points in this space shows that this species is a *climate generalist* in the sense that it can occur in most of Africa’s environmental range (Figure 3). Accordingly, its habitat is fairly widespread throughout Africa, with the main exceptions being the Sahara Desert, the Southern portion of South Africa, and parts of the Congo Basin (Figure 4A). This model was found to have AUC of 0.77 corresponding to sensitivity of 0.93 and specificity of 0.57 at its optimal accuracy of 0.75. Because these statistics were computed using background points in place of verified absences, these performance statistics represent a lower bound to accuracy. Analysis with boosted regression trees identified maximum December temperature, minimum September temperature, minimum March temperature, precipitation in November, and annual precipitation to be the key variables separating niche from non-niche habitats.

**Figure 5:**
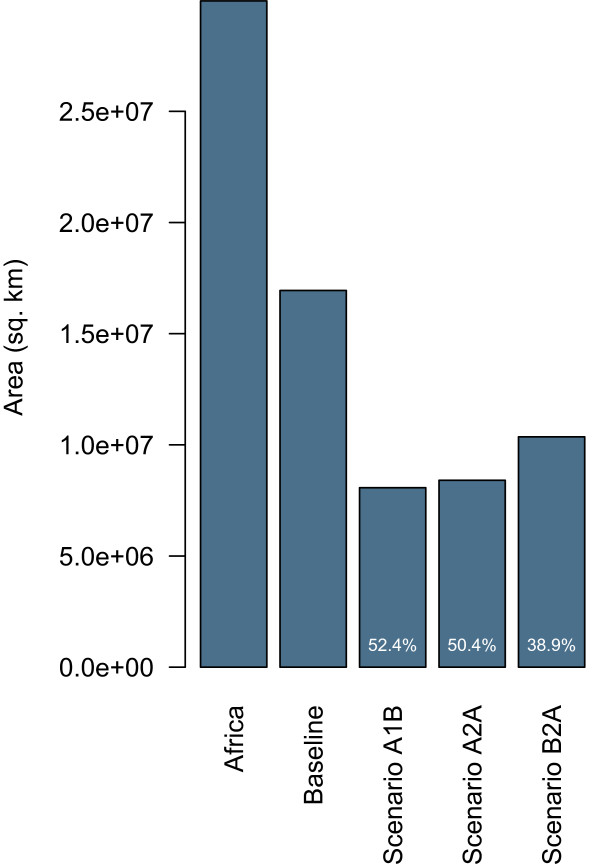
**Summary of the difference in current and projected total habitable area of *Anopheles arabiensis*.** Current distribution of *Anopheles arabiensis* habitat in Africa compared with the total land area of Africa and potential distribution under three climate change scenarios. Overplotted quantities are percent habitat loss from baseline.

**Figure 6:**
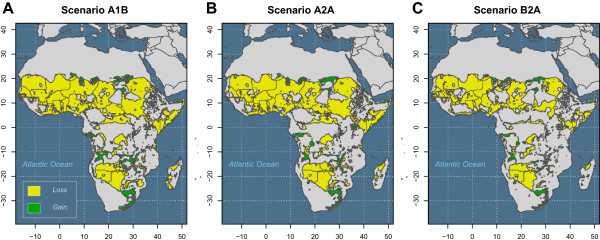
**Differences between the current and projected distribution of *Anopheles arabiensis*. A**. Losses and gains of *Anopheles arabiensis* habitat in Africa under future climate scenario A1B compared with the current distribution. **B**. Losses and gains of *Anopheles arabiensis* habitat in Africa under future climate scenario A2A compared with the current distribution. **C**. Losses and gains of *Anopheles arabiensis* habitat in Africa under future climate scenario B2A compared with the current distribution.

Under three plausible climate change scenarios, the future distribution of *An. arabiensis* is predicted to be considerably reduced (Figure 4). Figure 5 shows the total geographic area (sq. km) inhabitable by *An. arabiensis* under both baseline and forecasted future climate conditions and relative to the area of the entire Africa land mass. These results suggest that even in the absence of vector control and land conversion, the spatial distribution and total exposure of the African population to malaria transmitted by *An. arabiensis* is expected to change dramatically. Indeed, while the estimated effect of projected climate change is relatively large (reductions in area of 48%-61%), the differences among climate change scenarios are relatively small (Figure 4B-4D). Map differences between baseline and projected climate models suggest that reductions of habitat will be especially extensive in Western and Central Africa; portions of Botswana, Namibia, and Angola in Southern Africa; and portions of Sudan, South Sudan, Somalia, and Kenya in East Africa (Figure 6). The East African Rift Valley and Eastern Coast of Africa, where *An. arabiensis* is most abundant today, are expected to remain habitable. There will be some modest gains in habitat, especially on the margins of the current range in South Sudan, South Africa, and Angola. The key variables driving change in habitat were associated with temperature and precipitation from November to March. Particularly, maximum December temperature, maximum January temperature, precipitation in November, minimum March temperature, and minimum November temperature explain most of the difference between baseline and scenario A1B projections. All of these except minimum November temperature were important to the difference between baseline and scenario A2A and scenario B2A projections. Thus, it appears that the primary drivers of environmental change concern dry season climate, possibly interacting with the East African short rains.

**Figure 7:**
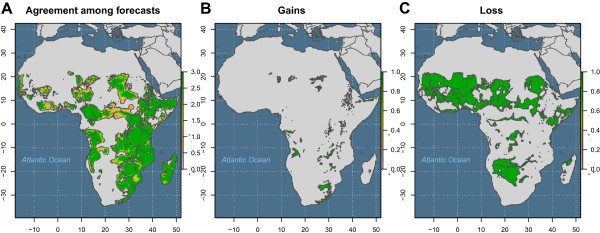
**Projected distribution of *Anopheles arabiensis *is robust to variations in climate change scenario. A**. Number of scenarios in which *Anopheles arabiensis* habitat is predicted to be lost or gained (grey: no scenario predicts habitat; orange: habitat predicted under one climate change scenario; light green: habitat predicted under two climate change scenarios; green: habitat predicted under all three climate change scenarios). **B**. Universal agreement among three climate change scenarios that *An. arabiensis* habitat will be gained. **C**. Universal agreement among three climate change scenarios that *An. arabiensis* habitat will be lost.

Given the inherent uncertainty about the effectiveness of present and future climate policies and the contingent scientific uncertainties that obtain at the present time, exactly what trajectory the future climate will take remains a major continuing unknown. An important question, therefore, is how sensitive are these projected gains and losses of *An. arabiensis* habitat to the details of the climate scenarios for which they are computed. To address this issue of the *robustness* of our projections, agreement among the three scenarios was calculated. First, for each pixel the number of scenarios for which it was predicted to be habitable by *An. arabiensis* was calculated (Figure 7A). Then, for those locations in which all scenarios were in agreement, gains (Figure 7B) were disaggregated from losses (Figure 7C). These results show that for those locations where a change of habitability is predicted, all three scenarios agree in the large majority of cases. Of these, the overwhelming majority predict loss of habitat.

## Discussion

This study showed *An. arabiensis* to be a climate generalist with widespread potential distribution in Africa. The map of the current potential distribution of *An. arabiensis* is in broad agreement with other published maps [29,30,49] disagreeing, as expected, primarily at the margins and extreme interior (*i.e.*, Congo and Ogooué basins). An ecological niche model for *An. arabiensis* projected on data from global climate simulations predicts that despite these wide tolerances, the potential distribution of *An. arabiensis* is likely to be reduced by 48%-61% by 2050 (Figure 5).

These results are important for interpreting the effectiveness of ongoing campaigns to eliminate malaria in several parts of Africa. First, not all reductions in malaria burden should be attributed to elimination campaigns. The signature of climate on *An. arabiensis* distribution that was detected here may at least partly explain other declines in prevalence in sub-Saharan Africa, for instance on Pemba and in mainland Tanzania [8,9]. Second, as indoor malaria-control activities, such as IRS and the distribution of ITNs, increase in effectiveness, one should expect a greater proportion of infections to be acquired out of doors. As a result there will be diminishing returns to increasing malaria elimination efforts. This does not mean that such increases would be unwarranted or ineffective. To the contrary, reducing such efforts short of complete elimination is an invitation for resurgence. The current results may be useful just insofar as they highlight regions in which malaria is most likely to be acquired from and maintained by *An. arabiensis*.

A long view of malaria elimination should therefore strategically consider the future climate of Africa and deploy interventions accordingly. Several strategies might be recommended. Because malaria is predicted to persist longest in those regions that remain habitable to *An. arabiensis* after climate change, it may be expected that these will be the most difficult regions from which to eliminate malaria, and that these will be the sources of any resurgence. From one point of view, these then are the regions that should be targeted most intensively with methods for reducing indoor biting, *i.e.*, ITNs and IRS. The rationale for this strategy is that to eliminate malaria requires reducing transmission everywhere (to remove sources for resurgence). However, to reduce transmission in regions where outdoor biting is considerable, *i.e.*, regions where *An. arabiensis* will persist, requires proportionately greater reductions in indoor biting to compensate. An alternative strategy considers the future potential distribution of *An. arabiensis* to be relatively minimal already. If elimination can be achieved outside this region, then subsequent effort might be best concentrated in a *cordon sanitaire* to limit the potential for reintroduction and resurgence. Finally, a hybrid strategy might exploit the fact that the predicted future potential distribution of *An. arabiensis* is both minimal and fragmented. Since fragmented populations are most vulnerable to extinction, it would possibly be most effective to try a mixed strategy: isolate core infected areas to prevent reinfection and break up regions of transmission at their most vulnerable points. In any case, it would seem advisable to consider the probable future distribution of *An. arabiensis* in the design of malaria elimination strategies. How to optimally combine these strategies remains, to our knowledge, an open problem.

Our work raises a number of questions about disease risk mapping. The introduction to this paper argues on conceptual grounds that the potential distribution of a vector species is better identified using boundary estimation techniques than methods for classification (*i.e.*, methods that aim to discriminate presence from absence points or occurrence points from the background distribution of environments). A simple numerical example in two dimensions shows why this is the case. The current study is the first major application of a new method, LOBAG-OC, for boundary estimation of ecological niche models. Although earlier results showed LOBAG-OC to perform comparably to other methods, such as MAXENT, and to be robust to several tuning parameters, how to optimize LOBAG-OC remains an important problem for further study.

Finally, not all determinants of a species habitat are captured by the climate variables encoded in the WorldClim data set. Although these results are expected to be robust with respect to the environmental variables that determine the range limits of *An. arabiensis*, these data and this model were not designed for and are not expected to explain local variation in abundance. The spatial resolution at which the current study was performed is therefore not suited to quantifying local variation in human exposure or health risk. Thus, additional regionally targeted modelling exercises will be of importance for tactically guiding interventions. Such models should make particular use of local information on human population density and land cover [28]. Our study, in contrast, was designed to produce a coarse-grained picture of the *An. arabiensis* distribution at the continental scale. Given the correspondingly grand scale of current investments in elimination, this scale may indeed be the most suitable to designing effective malaria policies.

## Conclusion

The future distribution of African malaria is predicted to be more dependent on the distribution of *An. arabiensis* and environmental variables than the current distribution. Further, presence-background methods for modelling species distributions may be expected to yield results with unnecessarily high Type I error, higher than that of suitably chosen boundary identification methods. One presence-only method, LOBAG-OC, predicts that the total area habitable to *An. arabiensis* will be reduced by 48%-61% by the year 2050 due to changes in the global climate system. Both the magnitude and spatial distribution of this reduction appear to be robust to the choice of climate scenario. How to maximally exploit this ecological relationship in malaria control and elimination is now an important question for research.

## Competing interests

The authors declare that they have no competing interests.

## Authors’ contributions

JMD developed the model and performed the analysis. JMD and JCB jointly wrote the manuscript. Both authors read and approved the final manuscript.
